# Anti-TNF-α therapy induced psoriasis in rheumatoid arthritis patients according to FDA postmarketing surveillance data

**DOI:** 10.1038/s41598-023-37010-6

**Published:** 2023-06-27

**Authors:** Haroutyun Joulfayan, Tigran Makunts, Ruben Abagyan

**Affiliations:** Skaggs School of Pharmacy and Pharmaceutical Sciences, University of California San Diego, La Jolla, CA USA

**Keywords:** Rheumatoid arthritis, Adverse effects, Skin diseases

## Abstract

Rheumatoid arthritis, RA, is a chronic autoimmune disease characterized by joint pain, tenderness, swelling, and stiffness. This disease affects nearly 1% of the world population. RA predominates in females and typically develops between the ages of 30 and 50 years. Common therapeutics for the treatment of RA include immune system suppressants such as tumor necrosis factor, or TNF, inhibitors. There is growing concern related to multiple clinical cases reporting an unexpected onset of psoriasis following the use of TNF inhibitors. This adverse event is counterintuitive since some tumor necrosis factor inhibitors are indicated for the treatment of plaque psoriasis. In this study, we analyzed over 880 thousand postmarketing safety reports from patients being treated for RA with a single therapeutic and provided evidence for a statistically significant association of psoriasis adverse events with TNF inhibitor use as compared to methotrexate. Additionally, we quantified the reported odds ratios and their 95% confidence intervals between four individual TNF inhibitors and found that the degree of association with psoriasis was variable among the drugs studied, with certolizumab pegol exhibiting the highest reported risk.

## Introduction

Rheumatoid arthritis (RA) is a common chronic autoimmune disorder primarily attacking the joints, affecting between 0.5 and 1% of the world population^1^. RA is characterized by joint pain, tenderness, swelling, and joint stiffness^2^. While the cause of RA is not certain, risk factors include genetics, smoking, obesity, sex, and age^2^. Similar to other autoimmune diseases, RA predominantly affects females, with a 2:1 to 3:1 female to male ratio^1^. While RA can develop at any age, typical onset occurs between 30 and 50 years^3^. RA is commonly treated with immunosuppressants such as methotrexate, hydroxychloroquine, sulfasalazine, or leflunomide, T-cell costimulatory inhibitors such as abatacept, interleukin-6 receptor (IL-6R) antibodies such as tocilizumab, Janus kinase (JAK) inhibitors, and tumor necrosis factor (TNF) inhibitors such as certolizumab pegol, adalimumab, golimumab, infliximab, and etanercept^4^.

T-cell costimulatory inhibitors prevent the activation of T-cells, which are believed to play a key role in the pathogenesis of RA. Abatacept, containing an extracellular domain of CTLA-4, accomplishes this by competitively binding the proteins CD80 and CD86, blocking their interaction with CD28^5^. Another important class of therapeutics in treating RA are IL-6 pathway inhibitors, which either inhibit IL-6 directly or block its receptor IL-6R^6^. Another class of therapeutics, JAK inhibitors, suppress cytokine-mediated signals by the JAK-STAT pathway^7^.

Anti-TNF treatments suppress the inflammatory response which results from the binding of TNF to its receptors^8^. Recently, there has been growing concern over TNF inhibitor-induced psoriasis, with many case reports detailing the onset of psoriasis in patients using TNF inhibitors, such as certolizumab pegol^9–11^. This relationship is seemingly paradoxical given that four of the five TNF inhibitors are indicated as a treatment for a type of psoriasis, plaque psoriasis: certolizumab pegol^12^, adalimumab^13^, infliximab^14^, and etanercept, which is a fusion of the TNF receptor and the IgG1-Fc part of an antibody^15^.

There have been postmarketing reports of incidents of new and worsening psoriasis in patients administered any of the four of the aforementioned TNF inhibitors^12–15^. These effects may be related to the cross-regulation of TNF and type-I interferons, including interferon alpha (IFN-α)^16^. TNF downregulates IRF7 and NF-κB pathways, which are responsible for IFN-α and TNF-α production in plasmacytoid dendritic cells^17^. Blocking the activity of TNF allows the production of IFN-α to increase^10^. Increased IFN-α has been associated with an increased risk of psoriasis^18^, possibly explaining the association between the use of TNF inhibitors and an increased risk of psoriasis. In this study, we analyzed over 880 thousand RA reports from FDA Adverse Event Reporting System (FAERS) and quantifed the evidence using the latest population scale dataset for a statistically significant association of psoriasis with TNF inhibitor use. Furthermore, we quantified the difference in the reported risk of developing psoriasis in RA patients between the TNF inhibitor cohorts.

## Methods

### FDA adverse event reporting system (FAERS)

The United States Food and Drug Administration Adverse Event Reporting System (FAERS) is a data repository which collects voluntary drug-related reports from healthcare professionals, consumers, and legal representatives. In cases where the adverse event (AE) is reported to the manufacturer, the manufacturer is required to forward the report to FAERS. At the time of the study, FAERS contained 17,392,666 AE reports collected from the first quarter of 2004 (which included historical reports since 1982) to the second quarter of 2022. The reports are available online: https://www.fda.gov/drugs/questions-and-answers-fdas-adverse-event-reporting-system-faers/fda-adverse-event-reporting-system-faers-latest-quarterly-data-files.

### Data preparation

FAERS reports are added and stored quarterly in a set of text files. Subsets of data are organized by specific report fields (demographics, drug, AE, outcome, etc.) and their respective case identifiers. The data format is not uniform and has been modified several times since its inception. Therefore, appropriate modifications have been introduced. Additionally, as the AE reports are collected from all over the world, the respective drug brand names are translated into the generic equivalents^19–21^.

### Study outcomes

The MedDRA dictionary version 25.1 was searched to define the measured study outcomes by higher level terms such as “immune associated conditions not elsewhere classified (NEC)” and “psoriatic conditions”. All of the psoriasis-associated preferred terms (PT) were used in the query. In order to avoid any indication-related confounding effects, psoriatic conditions associated with RA, such as psoriatic arthropathy, were excluded from the MedDRA PT list. The following PTs were used to define *psoriasis* in the analysis: erythrodermic psoriasis, guttate psoriasis, nail psoriasis, dermatitis psoriasiform, pustular psoriasis, and psoriasis.

### Cohort selection

Out of the total of 17,392,666 AE reports in FAERS, there were a total of 881,182 RA indication-containing reports, and for 663,922 of them, RA was listed as the only indication. Those records were further split by monotherapies and only reports by physicians, pharmacists and other healthcare professionals were included to avoid bias and increase clinical relevance. The final monoindication + monotherapy sets were the following: certolizumab pegol (n = 5168), adalimumab (n = 9221), golimumab (n = 2899), tocilizumab (n = 4819), abatacept (n = 7574), infliximab (n = 5579), rituximab (n = 2519), etanercept (n = 89543), tofacitinib (n = 10686), and methotrexate (n = 6142). Demographic analysis was performed for TNF inhibitors and methotrexate RA AE cohorts (Tables 1 and 2). The following psoriasis terms were included: erythrodermic psoriasis, guttate psoriasis, nail psoriasis, dermatitis psoriasiform, pustular psoriasis, and psoriasis. With these psoriasis type terms, the *psoriasis* AE report numbers were calculated for each drug cohort: certolizumab pegol (n = 98), adalimumab (n = 107), golimumab (n = 20), tocilizumab (n = 29), abatacept (n = 40), infliximab (n = 29), rituximab (n = 11), etanercept (n = 260), tofacitinib (n = 24), and methotrexate (n = 7). Disproportionality analysis was performed using reported AE frequencies to calculate reporting odds ratios (RORs). These numbers were used to calculate *psoriasis* reported frequencies. Methotrexate was selected as the control cohort due to its unique mechanism of action (MOA) as an immunosuppressant which inhibits the conversion of folic acid to folate cofactors, and common use as a monotherapy in RA.

### Demographic analysis

Sex (Table 1).

**Table 1:** The total number of TNF inhibitor reports and methotrexate reports in the combined RA cohorts separated by reported sex.

	TNF inhibitors	Methotrexate	χ^2^ value
Male total reports n, (%)	19,848, (17.66%)	1271, (20.69%)	0.44
Female total reports n, (%)	87,161, (77.54%)	4529, (73.74%)	0.20
Unspecified total reports n, (%)	5401, (4.80%)	342, (5.57%)	0.11
Total reports n, (%)	112,410, (100%)	6142 (100%)	–

The χ^2^ value was calculated for each row, excluding the total reports row, by comparing the percentages between each column in the row.

Age (Table 2).

**Table 2:** The total number of TNF inhibitor reports and methotrexate reports in the combined RA cohorts separated by reported age.

	TNF inhibitors	Methotrexate	χ^2^ value
< 30 y.o. n, (%)	3120, (3.52%)	92, (1.89%)	1.41
30–39 y.o. n, (%)	6448, (7.26%)	250, (5.13%)	0.88
40–49 y.o. n, (%)	13,263, (14.94%)	570, (11.70%)	0.90
50–59 y.o. n, (%)	26,504, (29.86%)	1111, (22.81%)	2.18
60–69 y.o. n, (%)	24,625, (27.74%)	1377, (28.28%)	0.01
> = 70 y.o. n, (%)	14,801, (16.68%)	1470, (30.18%)	6.04
Total reports n, (%)	88,761, (100%)	4870, (100%)	–

Records with unknown and invalid ages (< 1 y.o.) were excluded in order to minimize noise in the data. The χ^2^ value was calculated for each row, excluding the total reports row, by comparing the percentages between each column in the row.

### Statistical analysis

#### Descriptive statistics

**Figure 1:**
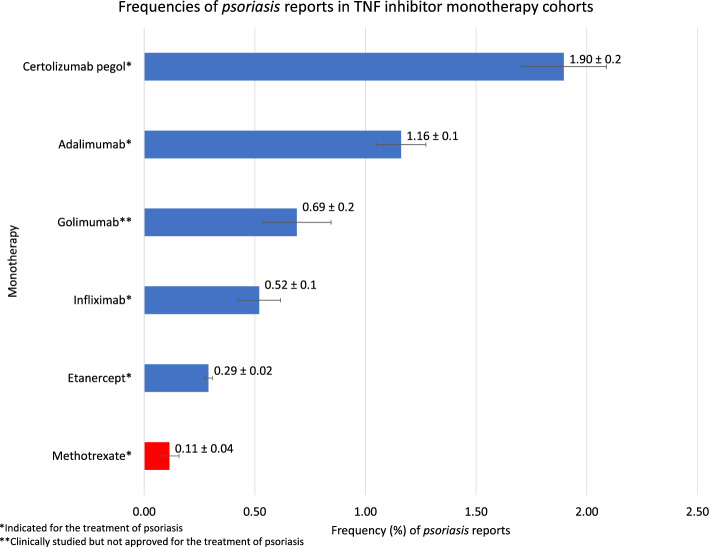
Frequencies of reported *psoriasis* AEs in RA patients undergoing TNF inhibitor monotherapy. Methotrexate is the control shown in red.

Frequencies for each studied side effect (Figs. 1, 3) was calculated by the equation:

1 $${\text{Frequency}} = \left( {{\text{nReports}}\,{\text{ with }}\,{\text{psoriasis}} \,{\text{in }}\,{\text{a}} \,{\text{cohort}}} \right)/{\text{nReports}}\,{\text{ in}} \,{\text{a}} \,{\text{cohort}}*100$$

Frequency error:

2 $${\text{Error}} = \left( {\sqrt {{\text{nReports}} \,{\text{with}}\, {\text{psoriasis}}\,{\text{ in}}\,{\text{ a}}\, {\text{cohort}}} } \right)/{\text{nReports}}\,{\text{ in}}\,{\text{ a}}\,{\text{ cohort}}*100$$

#### Comparative statistics

**Figure 2:**
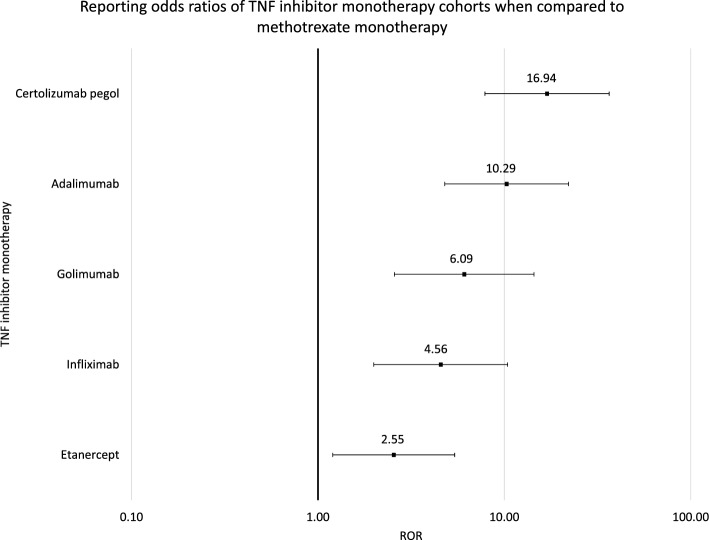
Reporting odds ratios were calculated by comparing the reported numbers of *psoriasis* reports and total reports between each TNF inhibitor monotherapy and the methotrexate cohort. The combined TNF inhibitors cohort exhibited a significant ROR of 4.03 with a 95% CI between 1.91 and 8.49. The ranges represent 95% CIs. The x-axis is shown in logarithmic scale.

Psoriasis report rates were compared via the Reporting Odds Ratio (ROR) analysis for Figs. 2, 4 and 5 and Tables 3, 4 and 5 using the following equations:

3 $$\mathrm{ROR}=(\mathrm{a}/\mathrm{b})/(\mathrm{c}/\mathrm{d})$$

where Number of cases in exposed group with psoriasis, Number of cases in exposed group with no psoriasis, Number of cases in control group with psoriasis, Number of cases in control group with no psoriasis.

**Table 3:** RORs and 95% CIs were calculated from comparisons between each TNF inhibitor monotherapy cohort and the methotrexate cohort.

Drug	ROR	95% CI
Certolizumab pegol versus methotrexate	16.94	[7.86, 36.50]
Adalimumab versus methotrexate	10.29	[4.79, 22.12]
Golimumab versus methotrexate	6.09	[2.57, 14.42]
Infliximab versus methotrexate	4.56	[1.99, 10.41]
Etanercept versus methotrexate	2.55	[1.20, 5.41]

**Table 4:** RORs and 95% CIs were calculated from comparisons between each studied monotherapy cohort and the methotrexate cohort.

Drug	ROR	95% CI
Certolizumab pegol versus methotrexate	16.94	[7.86, 36.50]
Adalimumab versus methotrexate	10.29	[4.79, 22.12]
Golimumab versus methotrexate	6.09	[2.57, 14.42]
Tocilizumab versus methotrexate	5.31	[2.32, 12.12]
Abatacept versus methotrexate	4.63	[2.07, 10.34]
Infliximab versus methotrexate	4.56	[1.99, 10.41]
Rituximab versus methotrexate	3.83	[1.48, 9.88]
Etanercept versus methotrexate	2.55	[1.20, 5.41]
Tofacitinib versus methotrexate	1.97	[0.85, 4.58]

**Table 5:** RORs and 95% CIs were calculated from comparisons between the certolizumab pegol monotherapy cohort and each of the other studied monotherapy cohorts.

Drug	ROR	95% CI
Certolizumab pegol versus adalimumab	1.65	[1.25, 2.17]
Certolizumab pegol versus golimumab	2.78	[1.72, 4.51]
Certolizumab pegol versus tocilizumab	3.19	[2.11, 4.84]
Certolizumab pegol versus abatacept	3.64	[2.52, 5.27]
Certolizumab pegol versus infliximab	3.70	[2.44, 5.61]
Certolizumab pegol versus rituximab	4.41	[2.36, 8.23]
Certolizumab pegol versus etanercept	6.64	[5.25, 8.39]
Certolizumab pegol versus tofacitinib	8.59	[5.49, 13.44]
Certolizumab pegol versus methotrexate	16.94	[7.86, 36.50]

4 $$\mathrm{LnROR}=\mathrm{Ln}(\mathrm{ROR})$$

Standard Error of Log Reporting Odds Ratio;

5 $${\mathrm{SE}}_{\mathrm{LnROR}}=\sqrt{1/\mathrm{a}+1/\mathrm{b}+1/\mathrm{c}+1/\mathrm{d}}$$

95% Confidence Interval;

6 $$95{\text{\% CI}} = \left[ {{\text{exp}}\left( {{\text{LnROR}} - 1.96 \times {\text{SE}}_{{{\text{LnROR}}}} } \right),{\text{exp}}\left( {{\text{LnROR}} + 1.96 \times {\text{SE}}_{{{\text{LnROR}}}} } \right)} \right]$$

## Results

### Methotrexate-treated patient cases as a control set

Methotrexate, an immunosuppressant drug, was selected as the control RA treatment based on its unique MOA as a competitive dihydrofolate reductase inhibitor^22^. Methotrexate monotherapy exhibited the lowest reported frequency of *psoriasis* at 0.11% out of all of the monotherapies studied. Methotrexate is a first-line therapeutic for RA in a clinical setting, making it a suitable control choice.

### TNF inhibitor-induced *psoriasis*

The reported frequencies of *psoriasis* AEs were calculated for each TNF inhibitor monotherapy. The frequencies of *psoriasis* reports between the TNF inhibitors ranged from 0.29 to 1.90%: certolizumab pegol 1.90%, adalimumab 1.16%, golimumab 0.69%, infliximab 0.52%, and etanercept 0.29% (Fig. 1).

The statistical significance of *psoriasis* association between the TNF inhibitor monotherapy cohorts and methotrexate cohort was calculated at 95% CI level: certolizumab pegol ROR 16.94 and 95% CI [7.86, 36.50], adalimumab 10.29 [4.79, 22.12], golimumab 6.09 [2.57, 14.42], infliximab 4.56 [1.99, 10.41], and etanercept 2.55 [1.20, 5.41]. The combined cohort of TNF inhibitors had an ROR 4.03 with a 95% CI of [1.91, 8.49] (Table 3, Fig. 2). The RORs and their 95% CIs demonstrate the risk of induced *psoriasis* in RA patients using TNF inhibitor monotherapies.

### Induced psoriasis using non-TNF inhibitor monotherapies

**Figure 3:**
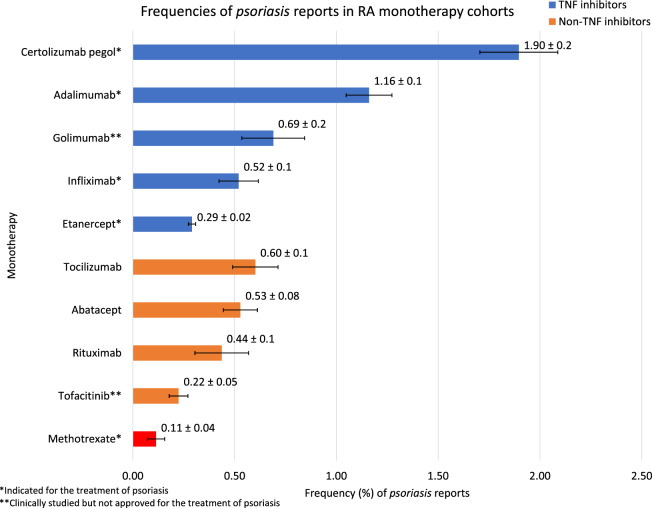
Frequencies of *psoriasis* reports in each of the studied cohorts. Methotrexate is the control shown in red.

**Figure 4:**
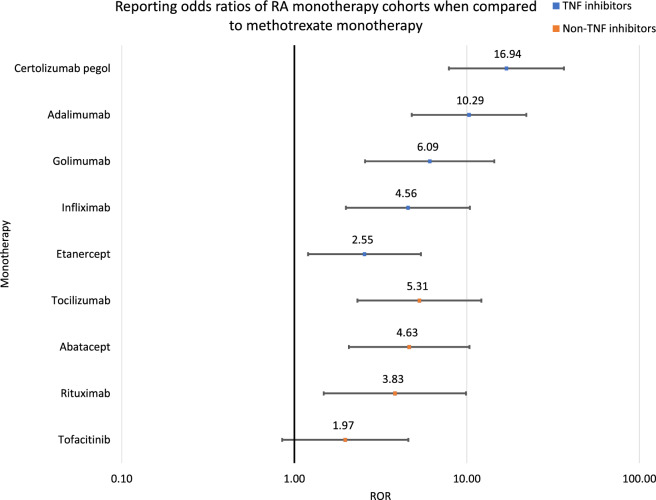
Reporting odds ratios were calculated for every monotherapy cohort by comparing the *psoriasis* reports and total reports between each monotherapy cohort and the methotrexate cohort. The ranges represent 95% CIs. The x-axis is shown in logarithmic scale.

To further expand on the evidence of RA therapeutic-associated *psoriasis*, we quantified the frequencies of *psoriasis* reports for other RA monotherapy cohorts: tocilizumab 0.60%, abatacept 0.53%, rituximab 0.44%, tofacitinib 0.22%, and methotrexate 0.11% (Fig. 3). All of the non-TNF inhibitor monotherapy cohorts, except tofacitinib, were significantly associated with *psoriasis* when compared to methotrexate: tocilizumab ROR 5.31 and 95% CI [2.32–12.12], abatacept 4.63 [2.07–10.34], rituximab 3.83 [1.48–9.88], and tofacitinib 1.97 [0.85–4.58] (Table 4, Fig. 4). The combined non-TNF inhibitor therapeutic reports, when compared to the methotrexate cohort, had ROR 3.58 and 95% CI of [1.66–7.69]. The RORs and 95% CIs of the non-TNF inhibitor cohorts demonstrate the statistically significant risk of developing *psoriasis* for RA patients using any of the studied monotherapy cohorts, except tofacitinib.

Tofacitinib was the only monotherapy cohort studied with a 95% CI that was not statistically significant (the 95% CI range overlapped with the ROR value of 1.00), whereas all of the other monotherapy cohorts had 95% CIs that were greater than 1.00 in both the lower and upper bounds.

The small number of *psoriasis* reports in the methotrexate-treated patient cases allowed for overlapping 95% CIs between the individual TNF inhibitors. In order to clarify the difference between the TNF inhibitors, we derived the ROR + 95% CI values by doing a pairwise comparison without methotrexate as a common control.

**Figure 5:**
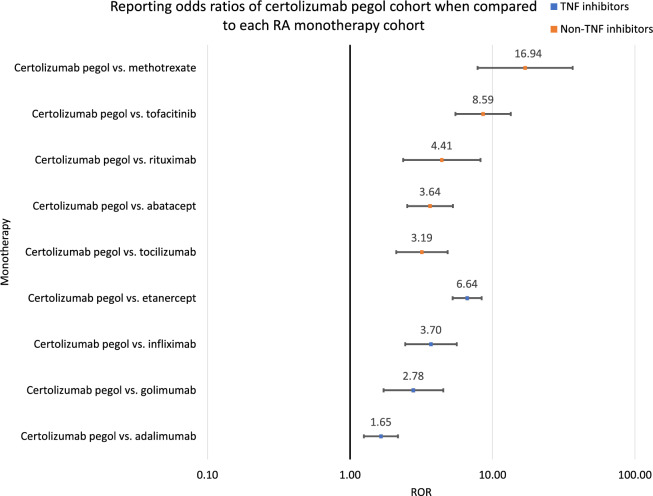
Reporting odds ratios were calculated for the association between certolizumab pegol and *psoriasis* by comparing the reported numbers of *psoriasis* reports and total reports between the certolizumab pegol cohort and each other RA monotherapy cohort. The ranges represent 95% CIs. The x-axis is shown in logarithmic scale.

Certolizumab pegol had a greater and statistically significant association with *psoriasis* AEs when compared directly with other RA drugs used as a control cohort instead of methotrexate. Certolizumab pegol compared to adalimumab yielded ROR 1.65 and 95% CI [1.25, 2.17], compared to golimumab 2.78 [1.72, 4.51], compared to tocilizumab 3.19 [2.11, 4.84], compared to abatacept 3.64 [2.52, 5.27], compared to infliximab 3.70 [2.44, 5.61], compared to rituximab 4.41 [2.36, 8.23], compared to etanercept 6.64 [5.25, 8.39], compared to tofacitinib 8.59 [5.49, 13.44], and compared to methotrexate 16.94 [7.86, 36.50] (Table 5, Fig. 5). It is noteworthy that the lower bound of the 95% CI range is greater than 1.00 for comparisons of certolizumab pegol with all of the other monotherapy cohorts.

## Discussion

In summary, we arrived at three sets of conclusions: (i) all TNF inhibitors have a statistically significant association with *psoriasis* in RA treated patients compared to the single methotrexate control group, (ii) not all of the non-TNF inhibitor treatments were associated with *psoriasis* compared to the single methotrexate control group, with some overlapping with the TNF inhibitor 95% CI ranges, (iii) pairwise comparisons of certolizumab pegol with both other TNF inhibitors and non-TNF inhibitor therapeutics show a greater and statistically significant association with *psoriasis*.

Thus, certolizumab pegol was found to have a particularly high reported risk of *psoriasis* AE when compared to all the other RA monotherapy cohorts studied.

To our knowledge, this is the first study to analyze over 660 thousand AE reports for RA indication in postmarketing data to quantify the association between TNF inhibitor monotherapy use and *psoriasis.* In addition, we quantified the risk of *psoriasis* in RA patients undergoing other types of therapeutic treatments such as IL-6 inhibitors (tocilizumab) and JAK inhibitors (tofacitinib), among others. We discovered that all TNF inhibitor treatments have a statistically significant association with *psoriasis* in RA patients. We also discovered that all of the non-TNF inhibitor cohorts, except tofacitinib, were statistically significantly associated with *psoriasis*, though to a lesser extent than certolizumab pegol. Tofacitinib was the only non-TNF monotherapy that was not statistically significantly associated with *psoriasis* within the 95% CI.

While using methotrexate as a single control for all of the monotherapies was convenient, there was an overlap of their 95% CI ranges, making it difficult to differentiate between the propensity of developing *psoriasis* for particular pairs of drugs. To address this issue, pairwise ROR + 95% CI analyses were performed. These results revealed that certolizumab pegol exhibited the highest reported risk, with 95% CI statistically separating it from all of the other studied monotherapies (Table 5, Fig. 5).

### Establishment of psoriasis AE dependencies on TNF inhibitor treatment

Given that RA and plaque psoriasis are both treated by many of the same TNF inhibitor therapeutics^12–15^, there may be co-occurrence of RA with psoriasis, independent of the treatment. However, this study excluded all non-RA indications during report selection to minimize any confounding effects. The large variability of reported *psoriasis* AEs between different RA monotherapy cohorts, ranging from certolizumab pegol 1.90% to methotrexate 0.11%, indicates that *psoriasis* AEs are treatment-dependent. These differences are further confirmed by more rigorous statistical comparison using 95% CIs between pairs of treatments. All of the TNF inhibitor monotherapy cohorts exhibited a significant association with *psoriasis* AEs, supporting the establishment of an association between TNF inhibitor therapeutic use and induced *psoriasis*.

### Uniquely high ROR of psoriasis AEs with certolizumab pegol compared to all other treatments

**Figure 6:**
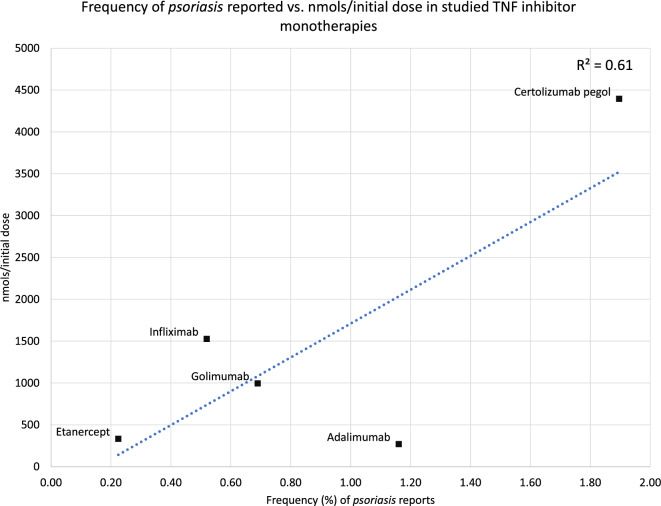
Scatter plot of the association between the frequency of *psoriasis* reports in the x-axis and the nmols/(initial dose) of the TNF inhibitor studied in the y-axis. The linear trendline, shown as a dotted blue line, and R-squared value, 0.61, are displayed.

Of the monotherapies studied, certolizumab pegol exhibited the highest reported frequency and ROR of *psoriasis* AEs when compared to the methotrexate cohort or any other treatment. Even when certolizumab pegol was compared to the second-highest *psoriasis*-risk associated RA drug, certolizumab pegol demonstrated a higher reported risk of *psoriasis* by 25% to 117% (Table 5). The higher frequency and ROR of certolizumab pegol may be due to the higher initial dosage^12^ recommended for certolizumab pegol compared to the other TNF inhibitors studied. In addition, certolizumab pegol is unique among the TNF inhibitors studied in that it is PEGylated, which is associated with a lower elimination rate^12^. A lower elimination rate, in addition to its higher initial dosage, may help to explain its higher risk of *psoriasis* AEs, even among the TNF inhibitor cohorts, given that TNF inhibition is correlated with psoriasis^23^. To determine whether more TNF inhibition may be associated with a higher frequency of *psoriasis* AEs, the nanomoles per recommended RA initial dose (nmols/(initial dose)) were calculated for each TNF inhibitor monotherapy cohort and compared with the respective cohort’s frequency of psoriasis reports (Table 6, Fig. 6).

**Table 6:** Calculated nmols/(initial dose) for each TNF inhibitor using its approximate molecular mass in g/mol and recommended initial dose for RA patients in milligrams (mg).

Drug name	Reported psoriasis frequency (%)	Approximate molecular mass (g/mol)	RA initial dose (mg) and administration	Estimated number of moles administered per dose (nmols/(initial dose))
Certolizumab pegol	1.90	91,000^12^	400 (SQ)^12^	4395.60
Adalimumab	1.16	148,000^13^	40 (SQ)^13^	270.27
Golimumab	0.69	150,500^23^*	149.82 (IV)^23^**	995.48
Infliximab	0.52	149,100^14^	227.73 (IV)^14^***	1527.36
Etanercept	0.29	150,000^15^	50 (SQ)^15^	333.33

*Golimumab contains many glycoforms and, as such, has a listed molecular mass of a range of approximately 150 to 151 kilodaltons^23^; in this analysis, we use the average between the two listed values for the approximate molecular mass in g/mol.

**The recommended RA initial dose for golimumab is 2 mg/kg of body mass. In this analysis, we calculated the average golimumab patient body mass from reported body masses in this study’s golimumab cohort, which is 74.91 kg, to determine the RA initial dose in mg.

***The recommended RA initial dose for infliximab is 3 mg/kg. In this analysis, we calculated the average infliximab patient body mass from reported body masses in this study’s infliximab cohort, which is 75.91 kg, to determine the RA initial dose in mg.

The estimated molar amounts [nanomoles] were calculated as follows: $\left( {{\text{initial}} \,{\text{dose}}\, {\text{of}}\, {\text{therapeutic }}\,{\text{in}} \,g} \right)/\left( {{\text{approximate}}\, {\text{molecular}} \,{\text{mass}} \,{\text{of}} \,{\text{therapeutic}} \,{\text{in }}\,{\text{g}}/{\text{mol}}} \right)*10^{9}$

The statistically significant associations of TNF inhibitors with *psoriasis* AEs were in line with current understanding of TNF inhibitors’ risk of psoriasis, as stated in the FDA package inserts of four of the five TNF inhibitors: certolizumab pegol^12^, adalimumab^13^, infliximab^14^, and etanercept^15^. However, given that golimumab’s package insert lacks a warning for psoriasis^24^, we underscore its statistically significant association with *psoriasis* as an adverse event.

Here we established and quantified an association of the use of TNF inhibitors, as well as the non-TNF inhibitors studied with the exception of tofacitinib, with the risk of *psoriasis* AEs.

## Conclusion

We established and quantified an association of TNF inhibitor monotherapies use by RA patients with psoriasis as an AE. All of the TNF inhibitor therapies approved for the treatment of RA and three out of the four non-TNF inhibitor drugs studied had a statistically significant association with *psoriasis* when compared to methotrexate. Out of the TNF inhibitors studied, certolizumab pegol exhibited the highest reported frequency of *psoriasis*.

### Limitations of this study

Due to the voluntary reporting nature of the FAERS system, the sample of reports in the FAERS database may be incomplete compared to real population data. FAERS reporting exhibits biases related to its newsworthiness and legal and scientific variables. Due to potential underreporting, some real-world data may be missing from the FAERS database which this study used to conduct its analyses. The changing criteria for AE diagnosis may lead to under or overreporting and affect the reporting frequencies. The frequencies and reporting odds ratios calculated are based on the sample of reports in the FAERS database and may not correspond to real population frequencies^25,26^. The individual cases were not adjudicated for causality assessment. Further studies may be required to establish the molecular mechanism behind this counterintuitive adverse event to better understand the uses and risks of TNF inhibitor therapies as it relates to RA and *psoriasis*. Most individual reports do not contain details on the progression of the AEs over time.

## Data Availability

The data sets available online to the public are de-identified. Institutional Review Board requirements do not apply under 45 CFR 46.102. https://www.fda.gov/drugs/questions-and-answers-fdas-adverse-event-reporting-system-faers/fda-adverse-event-reporting-system-faers-latest-quarterly-data-files. Both FAERS and AERS datasets are de-identified and are made available online at: http://www.fda.gov/Drugs/GuidanceComplianceRegulatoryInformation/Surveillance/AdverseDrugEffects/ucm082193.htm. Institutional Review Board Requirements do not apply under 45 CFR 46.102. There was no direct human participation in the study. All experiments were performed in accordance with relevant guidelines and regulations.
